# RPA and Rad27 limit templated and inverted insertions at DNA breaks

**DOI:** 10.1093/nar/gkae1159

**Published:** 2024-12-03

**Authors:** Yang Yu, Xin Wang, Jordan Fox, Qian Li, Yang Yu, P J Hastings, Kaifu Chen, Grzegorz Ira

**Affiliations:** Baylor College of Medicine, Department of Molecular and Human Genetics, One Baylor Plaza, Houston, TX 77030, USA; Department of Cardiology, Boston Children's Hospital, 300 Longwood Avenue, Boston, MA 02115, USA; Department of Pediatrics, Harvard Medical School, 25 Shattuck Street, Boston, MA 02115, USA; Kidney and Urinary Tract Center, The Abigail Wexner Research Institute at Nationwide Children's hospital, Columbus, OH 43215, USA; Baylor College of Medicine, Department of Molecular and Human Genetics, One Baylor Plaza, Houston, TX 77030, USA; Baylor College of Medicine, Department of Molecular and Human Genetics, One Baylor Plaza, Houston, TX 77030, USA; Department of Cardiology, Boston Children's Hospital, 300 Longwood Avenue, Boston, MA 02115, USA; Department of Pediatrics, Harvard Medical School, 25 Shattuck Street, Boston, MA 02115, USA; Baylor College of Medicine, Department of Molecular and Human Genetics, One Baylor Plaza, Houston, TX 77030, USA; Department of Cardiology, Boston Children's Hospital, 300 Longwood Avenue, Boston, MA 02115, USA; Department of Pediatrics, Harvard Medical School, 25 Shattuck Street, Boston, MA 02115, USA; Baylor College of Medicine, Department of Molecular and Human Genetics, One Baylor Plaza, Houston, TX 77030, USA

## Abstract

Formation of templated insertions at DNA double-strand breaks (DSBs) is very common in cancer cells. The mechanisms and enzymes regulating these events are largely unknown. Here, we investigated templated insertions in yeast at DSBs using amplicon sequencing across a repaired locus. We document very short (most ∼5–34 bp), templated inverted duplications at DSBs. They are generated through a foldback mechanism that utilizes microhomologies adjacent to the DSB. Enzymatic requirements suggest a hybrid mechanism wherein one end requires Polδ-mediated synthesis while the other end is captured by nonhomologous end joining (NHEJ) or by alternative end joining (Alt-EJ). This process is exacerbated in mutants with low levels or mutated RPA (*rtt105*Δ; *rfa1*-t33) or extensive resection deficiency (*sgs1*Δ *exo1*Δ). Templated insertions from various distant genomic locations also increase in RPA mutants as well as in *rad27*Δ and originate from fragile regions of the genome. Among complex insertions, common events are insertions of two sequences, originating from the same locus and with inverted orientation. We propose that these inversions are also formed by microhomology-mediated template switching. Together, we propose that a shortage of RPA, typical in cancer cells, may be a factor that stimulates the formation of templated insertions.

## Graphical Abstract

**Graphical Abstract:**
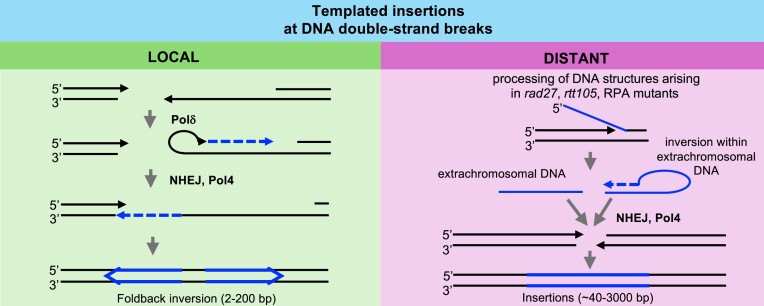

## Introduction

Templated insertions are a type of genome rearrangement wherein a segment of DNA is inserted into a chromosome, thereby duplicating existing genetic material. These types of copy number variations (CNVs) can lead to genetic disorders and are among the most common in cancer genomes (1,2). The inserted DNA may originate from nearby locations on the same chromosome (called hereafter local insertions) or from distant locations on the same or different chromosome (distant insertions). Templated insertions can be complex, involving multiple fragments copied from different parts of the genome (1,3). The size of templated insertions ranges from a few nucleotides to over a megabase. Multiple mechanisms have been proposed to explain templated insertions. Analysis of the junctions has demonstrated 0 to 20 nt of microhomologies, implicating several DNA double-strand break (DSB) repair pathways: nonhomologous end joining (NHEJ), alternative-end joining (Alt-EJ) and microhomology-mediated break-induced replication (MMBIR). In MMBIR, DSB ends can initiate low processivity, template switch-prone DNA synthesis at microhomologies (4,5). In a related model, one DSB end primes DNA synthesis while the other DSB end is captured by NHEJ, restoring chromosomal integrity (6). Support for such a hybrid mechanism comes from observations that DSB end mobility, Polδ, and NHEJ proteins are all necessary for at least some of the templated insertions at CRISPR/Cas9 induced events observed in cancer cells (6). However, a large fraction of the described events carry no microhomology on either DSB end, suggesting the involvement of a different polymerase than Polδ that requires initial microhomology, or that another mechanism contributes (6). A third proposed mechanism involves the capture of extrachromosomal DNA fragments at DSBs by NHEJ (3,7–9). The evidence supporting this mechanism includes microhomology typical for NHEJ at both ends of templated insertions and the requirement for key NHEJ enzymes. Various types of DNA can be captured at DSBs, including mitochondrial DNA (mtDNA), transposon cDNA and nuclear genomic DNA, as well as transformed single-stranded (ssDNA) or double-stranded DNA (dsDNA). Multiple fragments can be captured at DSBs at once, including a mixture of genomic DNA, mtDNA and transposon DNA, as reported in yeast, humans or plants (7,10–15). Additionally, Alt-EJ can result in very short local direct or inverted templated insertions, typically ranging from about 2 to 35 base pairs (bp) (16,17). These events require both PolQ and Polδ exonuclease activity to trim terminal nonhomology (18). Additionally, some models propose that specific inverted templated insertions can originate without any initial DSB, but rather from template switches within replication forks followed by extrusion of inverted ssDNA and its replication and reintegration into the genome (19).

Although the analysis of cancer genome sequences has provided a wealth of information about templated insertions, the mechanisms leading to their formation can only be inferred (e.g. (1,20)). One experimental approach to model templated insertions and identify enzymes or conditions that upregulate these events is through the sequencing of DSB repair products carrying rare, templated insertions. Templated insertions have been observed at HO endonuclease or CRISPR/Cas9 induced DSBs in yeast and humans, as well as at programmed DSBs at the V(D)J locus (3,6,9,21). Most of these events in yeast and humans are ∼50–300 bp in size. In wild-type yeast cells grown under optimal conditions, such insertions are very rare. However, in stressed stationary phase cells, they increase dramatically (9). The insertions from genomic DNA, mtDNA or transposon cDNA increase by ∼100–2000-fold, with the mtDNA and cDNA being controlled by yeast EndoG, Nuc1 (9). Templated insertions also increase in cells deficient in the 5′ flap endonuclease and helicase, Dna2. The function of Dna2 in limiting such insertions is conserved from yeast to humans and can be related to its role in processing overreplicated DNA at reversed replication forks or long 5′ flaps (3,22–24). In support of this possibility, it was recently demonstrated that overreplicated DNA at reversed replication forks can recombine across the genome, generating duplications (25). Genomic insertions observed in stationary phase cells or in *dna2*Δ mutants are not associated with deletion of the donor fragment, thus indicating a copy-paste mechanism. Inserted DNA in yeast and humans often originates from fragile regions of the genome, including telomeric and other repetitive regions, or R-loops (3,21). Microhomology-mediated templated insertions, including local inversions, have also been observed in other DSB inducible recombination assays. In these assays, regular homology driven repair was interrupted and transiently switched to microhomology driven synthesis (e.g. (26–28)).

In this study, we investigated the role of several DNA repair/replication enzymes in the regulation of templated insertions at DSBs. Specifically, we focused on proteins previously shown to regulate microhomology-mediated annealing of ssDNAs, including RPA and the RPA regulatory factor Rtt105 (29–33). Deficiency in the RPA complex is commonly observed in cancer cells experiencing replication stress, and it leads to various types of genome instability (34–36). In this study, we also investigated the role of enzymes functionally related to Dna2: the 5′ flap endonuclease Rad27, which, similar to Dna2, processes 5′ flaps during replication, and the Sgs1 and Exo1 enzymes, which promote extensive resection along with Dna2 (24,37). RPA and *rad27*Δ mutants exhibited increased levels of templated insertions and displayed specific and distinct patterns of insertions. Furthermore, we characterized different mechanisms of local and distant *de novo* templated insertions with inverted orientation. Based on our findings in the yeast model system, we propose that a shortage of RPA in cancer cells is one possible factor stimulating the formation of microhomology-mediated templated insertions.

## Material and methods

### Yeast strains

All yeast strains are JKM139 derivatives (38) (*MAT***a** DELho *hml*::*ADE1 hmr*::*ADE1 ade1 leu2-3 112 lys5 trp1*::*hisG ura3-52 ade3*::*GAL10*::*HO*) and are listed in Supplementary Table S1. All strains were constructed by transformation. The *rfa1*-t33 mutant was constructed using CRISPR/Cas9 by transforming the plasmid bYY37 carrying gRNA sequence (ATGGCAGCAACAGAACCTTC) with the plasmid bRA90 (a gift from James Haber) as the backbone and ‘rfa1-S373P’ oligo (CCTATGGAATCAGCAAGCCCTTGATTTCAACCTTCCTGAAGGTCCAGTTGCTGCCATTAAAGGTGTTCGTGTGACGGATTTTG) as the template.

### Yeast growth and DSB induction

Yeast cells were grown overnight in YPD (1% yeast extract, 2% peptone, 2% dextrose), washed twice with YP-raffinose (1% yeast extract, 2% peptone, 2% raffinose) media, inoculated into 10 mL YP-raffinose media, and incubated at 30°C overnight. When the cell density was about 2 × 10^7^/mL, 500 μL cells were spread onto YP-galactose (1% yeast extract, 2% peptone, 2% galactose, 2.5% agar) plates to induce HO expression and the DSB at *MAT***a** locus, and incubated at 30°C for 5 days. Each sample was spread on 5–7 150 × 15 mm YP-galactose plates. The number of colonies was counted. All colonies on YP-galactose plates were collected, combined, and mixed vigorously. Approximately 80 μL cells were spun down for isolating genomic DNA.

### Amplicon sequencing of DSB repair products by *Break-Ins*

Amplicon sequencing was performed as previously described (9). Standard glass beads-phenol/chloroform/isoamyl alcohol (25:24:1) method was used for isolating genomic DNA. The DNA was dissolved in water and adjusted to a concentration of 10 ng/μL. The construction of the amplicon sequencing library was adapted from Illumina's protocol #15 044 223 Rev. B. Briefly, the sequencing libraries were constructed with two rounds of PCR using KAPA HiFi HotStart ReadyMix polymerase (Roche, 7 958 927 001). For the first round of PCR, 3.2 μL genomic DNA and 0.5 μL 10 μM primers were used for a total volume of 25 μL. The forward and reverse primers targeting the *MAT***a** locus are located 11-bp upstream or 18-bp downstream of the HO cleavage site respectively, containing Illumina adapter sequence and 3 bases unique home index as described (9). The thermocycling conditions were: 95°C for 5 min; 22 cycles of 98°C for 20 s, 65°C for 30 s, 72°C for 3 min; 72°C for 10 min. After the first round of PCR, 18 μL PCR product was purified with an equal volume of AMPure XP beads (Beckman Coulter, A63880). The DNA was eluted with 52.5 μL of 10 mM Tris (pH 8.5) and used as the templates for the second round of PCR. For the second round of PCR, 5 μL template DNA, 5 μL index primer N7xx and 5 μL index primer S5xx from Nextera XT V2 Index kit (Illumina, FC-131–2001) were used for the total volume of 50 μL. The following conditions were used for the second round of PCR: 95°C for 5 min; 8 cycles of 95°C for 30 s, 55°C for 30 s, 72°C for 3 min; 72°C for 10 min. Then, the PCR product was purified with 1.2x volume of AMPure XP beads and eluted with 10 mM Tris pH 8.5. The average size of each sample was measured with TapeStation and the DNA concentration was measured with Qubit dsDNA BR Assay Kit (ThermoFisher, Q32850). Then, the DNA was diluted to a concentration of 4 nM. An equal volume of DNA from ∼20 samples was pooled into the library. The 0.2 M NaOH was used to denature the pooled library and PhiX library (Illumina, FC‐110‐3001). After diluting the libraries with pre-chilled HT1 to 12 pM, pooled library and PhiX library were mixed with the ratio 9:1, further denatured by incubating at 96°C for 2 min and immediately put in an ice-water bath for 5 min. The MiSeq and Reagent Kit v3 (600 cycles) (Illumina, MS-102–3003) were used for sequencing. The cluster density for all libraries was ∼1100 K/mm^2^.

### Analysis of insertion of transformed DNA

The 69-mer reverse-phase cartridge-purified oligo ordered from Sigma-Aldrich was dissolved to 100 μM in TE buffer. The oligo CATTGAACAACATGTTGCTGTAAGNN NNACTCATAGTACAGACGGCGTGTATCTGGTATTGGTCTGTAC contains 4-mer random nucleotides in the middle (NNNN), 8-nt inverted repeats (underlined) at the 3′ terminus with an 18-nt spacer. To analyze the insertions of transformed DNA, 10 mL cells were collected when the density reached ∼1.5 × 10^7^ cells/mL in YEP-Raffinose, washed twice with water and mixed with 240 μL 50% PEG3350, 36 μL 1M lithium acetate, 40 μL 100 μM DNA and 44 μL water to a total of 360 μL. After incubating at 30°C for 30 min followed by 42°C for 30 min, the cells were spun down, resuspended in 4.2 mL water, and spread on 150 × 15 mm YEP-Galactose plates (700 μL per plate). A total of 6 plates were spread. The plates were incubated at 30°C for 5 days. All colonies on YP-galactose plates were collected, pooled and mixed vigorously. Approximately 80 μL cells were spun down for isolating genomic DNA for amplicon sequencing as above.

### Detection of insertions from *Break-Ins* analysis

The identification of insertions is outlined in the iDSBins protocol published previously (9) (https://github.com/gucascau/iDSBins.git). Briefly, we developed iDSBquality pipeline for raw reads preprocessing using BBDuk to eliminate Phix reads and to ensure reads quality control. Subsequently, the iDSBdetection pipeline was used, integrating PEAR and BLASTN alignments for the detection of reads featuring >10-bp insertions at DSB. Leveraging the information obtained from BLASTN alignments, we introduced iDSBdeduplication to effectively eliminate redundant reads. The identification of junction indels and microhomologies, as well as the donor number and annotation were accomplished through iDSBannotation. These tailored protocols collectively constitute a comprehensive approach for the systematic identification and characterization of large insertions at DSBs.

### Feature analysis of large insertion

The feature analysis of large insertions, including R-loop, ARS, telomere, tRNA and tandem repeats, was performed using the package LargeInsertionFeature (https://github.com/gucascau/LargeInsertionFeature.git). Briefly, random control locations were generated by shuffling the real observed locations of the insertions using the algorithm BEDTools shuffle. Bedtools v2.253 was used to retrieve genomic features that overlapped with or were located close to the insertion donor sequences in the genome.

### Detection of local foldback *MAT*a insertions and distant inverted insertions

**Figure 1.**
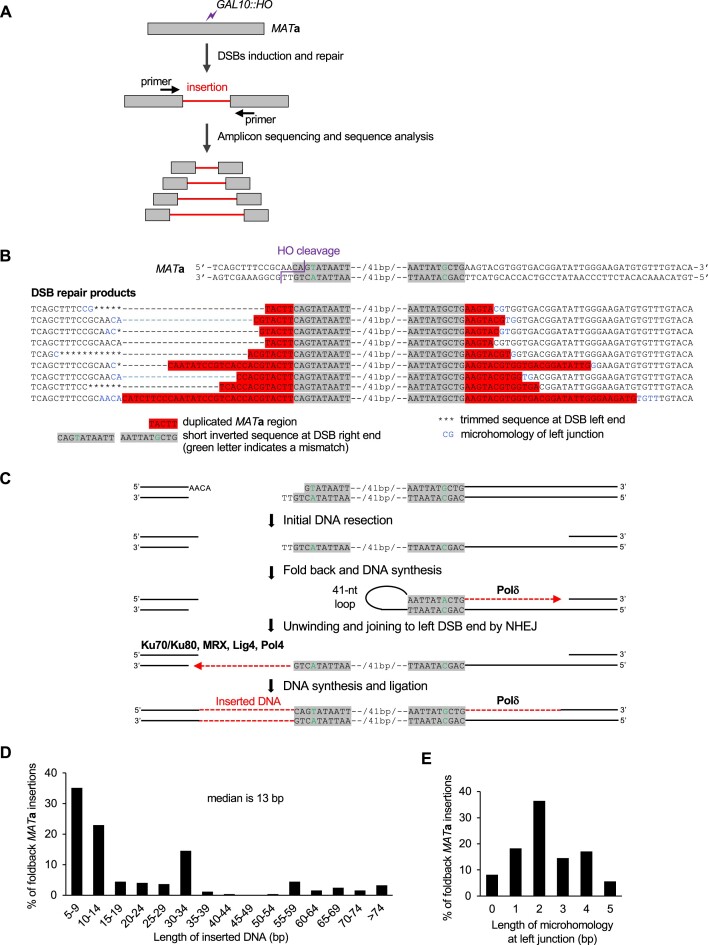
Local foldback inverted insertions at DSBs. **(A)** Scheme of the *Break-Ins* method for detecting insertions at DSBs. DSBs are induced by the HO endonuclease at the *MAT***a** locus. The DSB repair products are pooled and subjected to amplicon sequencing using the MiSeq platform. The analysis focuses on reads with >10-bp insertions at DSBs. **(B)** The inverted duplication from the region close to the HO cleavage site. Upper, the double strand *MAT***a** sequence including HO cleavage site. The HO induces DSBs with 4-nt 3′ overhangs. Lower, examples of the inverted insertions in DSB repair products copying the sequence mediated by foldback 10-bp imperfect inverted microhomology sequences with a 41-nt spacer. The sequencing data of wild-type cells are taken from a previous publication (9). **(C)** Model presenting the mechanism of local foldback inversions at DSBs. The right end of HO-induced DSBs is resected to expose 10 nt imperfect inverted microhomologies marked with grey. The ssDNA then folds back to form a hairpin with 41 nt loop, and Polδ synthesizes a short DNA. The newly synthesized DNA unwinds from the template and ligates to the left DSB via NHEJ. The other strand is synthesized and ligated. **(D)** Size analysis of the foldback inversions from WT, *sgs1*Δ *exo1*Δ, *rfa1*-t33 and *rtt105*Δ mutants combined. Foldback inversions shorter than 5 bp were not included. **(E)** Length of microhomology at left junction from WT, *sgs1*Δ *exo1*Δ, *rfa1*-t33 and *rtt105*Δ mutants combined. Foldback inversions shorter than 5 bp were not included.

To identify local templated foldback inversions, we initially delineated the template-inverted sequence based on two inverted 10-bp long microhomologies next to the telomere-proximal DSB end. The seed sequence, consisting of the first 5 bp copied (TACTT, Figure 1B, first foldback inversion example) followed by a short *MAT***a** sequence corresponding to inverted microhomology (CAGTATA or CAGCATA), was used for scanning sequencing reads for foldback inversion. By constraining this seed sequence (TACTTCAGTATA or TACTTCAGCATA), we ensured selection of foldback inversions of 5 bp or longer. Subsequently, the scanning process extended across both PEAR merged reads and unmerged reversed reads. As occurrences were not detected in unmerged reads, our analysis exclusively focused on merged reads for subsequent analyses. Utilizing BWA mem, we mapped these reads against chromosome III, in which the *HML* and *HMR* regions have been masked. To accurately deduplicate *MAT***a** reads, analysis of CIGAR strings was performed, with special emphasis on avoiding sequencing errors at both sides of *MAT***a** regions. Insertions (I) or deletions (D) at either MAT-L (left junction) or MAT-R (right junction) regions due to sequencing errors were disregarded. Following this, we executed deduplication, measuring insertion size and read count supporting each *MAT***a** hotspot insertion. Duplicated reads were identified as those sharing identical sequences containing the inverted sequence and their upstream and downstream 5 bp. Irrespective of read number, each local foldback inversion sequence was counted as a single event. To determine the 2 bp foldback inversions, we specifically selected reads corresponding to this 2 bp, but not longer, inversions. To identify distant inversions, we screened the reads corresponding to complex insertions that contained two donor sequences from a single locus but with reverse orientations.

### Analysis of insertions originating from transformed ssDNA

Raw reads were pre-processed with quality control and merged with PEAR v0.9.11 as described above. Reads with any substring of transformed ssDNA (e.g. CATTGAACAA, CAACATGTTG, TTGCTGTAAG, ACTCATAGTA, GTGTATCTGG, TACCAGATAC, TACTATGAGT or CTTACAGCAA) were recognized as insertions. Barcode sequences were defined between 5-bp upstream and 5-bp downstream of the barcode sequence. Perfect matches were required for these seed sequences. Thereafter, UMI features were used to distinguish between insertions with two identical and two different barcodes.

## Results

### Formation of local foldback inversion at DSBs

To study templated insertions in yeast, we employed an HO endonuclease-inducible system in which a single DSB per genome is induced at the *MAT***a** locus. This DSB cannot be repaired by homologous recombination because the strain lacks a homologous template, and both sister chromatids are cut by HO endonuclease. Instead, the DSB is repaired mostly by NHEJ (38), occasionally altering the cleavage site. An amplicon sequencing-based method, *Break-Ins*, described previously, was used to sequence DSB repair products (9) (Figure 1A). Analysis was limited to the sequencing reads that carried insertions of > 10 bp at DSBs among all repair products. Previous analyses have focused on the transfer of mtDNA and transposon cDNA to the nucleus, as well as the function of Nuc1 in the degradation of these non-nuclear DNA species (9). Our aim here was to gain a better understanding of insertions originating from the nuclear genome. Analysis of over 100 000 independent DSB repair products in wild-type cells revealed local insertions originating from the right side of the DSB that varied in length from 10 to over 150 bp. All these insertions exhibited an inverted, imperfect 10 bp microhomology at the junction. This inverted 10-bp sequence naturally occurs just 2 and 53 bp from the right DSB end and is separated by 41 bp (Figure 1B). We propose that resection of the right DSB end exposes two inverted repeats that anneal to each other to form a hairpin with a loop of 41 nt. After trimming of the terminal 2 nt at the 3′ end, new DNA synthesis is followed by displacement of the nascent strand that is then ligated to the second end of a DSB (Figure 1C). We further screened the same sequencing reads for even shorter insertions from this locus and identified many more insertions of 5–9 bp (Figure 1B,D). We note that identical sequencing reads corresponding to foldback inversion in a single experiment were considered as single event; however, it is possible that multiple identical events occurred independently. Also, we cannot exclude PCR or sequencing bias and therefore some events are either underrepresented or not sequenced. Finally, it is possible that 1–4-bp insertions form by the same mechanism, but these are difficult to distinguish from other mechanisms during NHEJ. The left DSB end and the inverted sequence shared only 0–5 bp microhomology (median = 2 bp) indicating that the repair was completed by regular NHEJ (Figure 1E). In conclusion, we observed very short foldback inversions formed at DSBs, much shorter than the ones previously reported (e.g. (39)). Also, the mechanism of their formation is different, as explained below.

### NHEJ and Polδ are required for the formation of local foldback inversions

**Figure 2.**
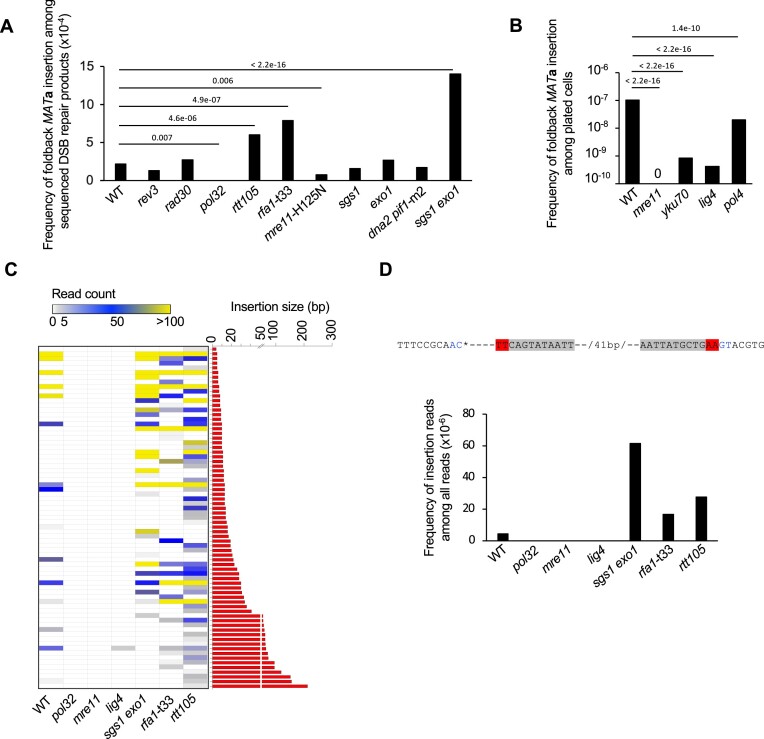
Genetic requirements for local foldback inversions at DSBs **(A)** Analysis of foldback inversions in indicated mutants. Foldback inversions shorter than 5 bp were not included. *P* values were determined using χ^2^ test; n – number of DSB repair products tested for each mutant is shown in Supplementary Table S2. **(B)** Foldback inversions in NHEJ mutants. P values were determined using χ^2^ test, n – number of cells plated is shown in Supplementary Table S2. **(C)** Size and read number distribution for foldback inversions in indicated mutants. Each row represents a specific insertion event. Some of the events have the same size but different left junctions. **(D)** Number of reads corresponding to a 2-bp foldback inversion in indicated mutants. The upper is the sequence of inverted insertion, and inverted microhomology is as shown in Figure 1B.

To investigate whether the formation of foldback inversions involves the proposed hybrid mechanism (Figure 1C), wherein one DSB end is extended by DNA synthesis at the hairpin, and the resulting duplicated region is unwound and captured by the second DSB end via NHEJ, we conducted an analysis of the genetic requirements for these events. First, we searched for the DNA polymerase involved in the DNA synthesis step. We found that elimination of Rev3 (Polζ) and Rad30 (Polη) had no effect on these events (Figure 2A, Supplementary Table S2). However, elimination of Pol32, a component of Polζ and a nonessential component of Polδ needed for BIR (40), completely eliminated foldback inversions. Since *rev3*Δ showed no phenotype, we conclude that Polδ, rather than Polζ, is responsible for the extension of microhomology-mediated foldback structures. This finding is consistent with previous reports highlighting the important role of Polδ in stabilizing microhomologies and hairpin structures (28,31,41). Secondly, to assess the contribution of NHEJ in foldback inversion events, we analyzed rare DSB repair events in NHEJ-deficient mutants *yku70*Δ, *lig4*Δ and *mre11*Δ. To increase the number of rare Alt-EJ events, >10-fold more cells were plated in these mutants. Our analysis revealed that Yku70, Lig4, or Mre11 were nearly essential for the occurrence of foldback inversions (Figure 2B). Three remaining events likely resulted from Alt-EJ and exhibited 3- or 4-nt microhomology at the DSB end used to capture duplicated sequences. We note that primers used to amplify the repaired locus are located very close to DSB ends (11 and 18 bp); therefore Alt-EJ events involving trimming of DSB ends could not be analyzed using this experimental design. Finaly, Pol4 that is needed for most of the insertions of mtDNA, retrotransposon cDNA, and from the nuclear genome in Dna2 deficient cells or in stationary phase cells (3,9), was also important for local inverted insertions. This is consistent with the primary role of Pol4 in NHEJ and Alt-EJ that require short DNA synthesis (9,42,43). Overall, our findings support the view that both Polδ dependent DNA synthesis and NHEJ or Alt-EJ are involved in the formation of very short foldback inversions.

### Rtt105, RPA and extensive resection suppress local foldback inversions

Next, we analyzed foldback events in RPA mutants, which were previously shown to affect microhomology-mediated repair (29–33). We tested the RPA mutant strain *rfa1*-t33, which exhibits decreased DNA binding, decreased growth, and defective replication (44–46) as well as the *rtt105*Δ mutant, which has a decreased level of nuclear RPA and reduced binding of RPA to ssDNA (33,47,48). The frequency of local foldback inversions mediated by microhomology among sequenced DSB repair products increased in both the *rtt105*Δ and *rfa1*-t33 mutant strains. (Figure 2A,C). We note that increased frequency of inverted insertions of 30–34 bp was observed in all mutants tested and is likely related to longer microhomology (AACA) matching the second end of the break (Supplementary Figure S1A). Previous studies have shown increased foldback inversions in the absence of Mre11 nuclease, which is known to process hairpin structures. Mre11 nuclease can process hairpin structures with short loops of up to approximately 8 nt and inhibits foldback inversions (39,49). Hence, the 41-nt loop here (Figure 1B,C) is unlikely to be processed by Mre11. Indeed, we found no increase in foldback inversions in the *mre11*-H125N nuclease mutant, consistent with Mre11 having no role in the cleavage of this hairpin loop (Figure 2A). Other proteins preventing foldback inversions are Rad1 nuclease and Tel1 kinase (50,39). To investigate the potential roles of these proteins in the formation of short, inverted insertions, we constructed *tel1Δ* and *rad1Δ* derivatives of *rtt105Δ*, which exhibit higher levels of such events and thereby facilitate testing. Elimination of Rad1 had no impact on inverted insertions in *rtt105*Δ while *tel1*Δ decreased these events significantly (Supplementary Figure S1B). Thus, Tel1 and Mre11 nuclease do not suppress inverted insertions as observed in other systems, but rather promote it. This could be related to their role in initial resection (51,52). Additionally, we investigated the role of extensive resection enzymes Sgs1, Dna2 and Exo1 in short foldback inversions. We observed an increase in local foldback inversions among NHEJ products in the *sgs1*Δ *exo1*Δ mutant, while no change was seen in the single mutants *sgs1*Δ, *exo1*Δ or *dna2*Δ *pif1-m2* (Figure 2A,C). We note that a similar increase is observed when inverted insertions are calculated per number of all cells plated and normalized by viability of *rtt105*Δ and *sgs1*Δ *exo1*Δ mutant cells (Supplementary Figure S1C). The MRX complex, along with Sae2, generates a few hundred nucleotides of ssDNA (37,53), which is sufficient to expose two inverted repeats located within the first 63 bp of the break. Without extensive resection, very short ssDNA may limit RPA binding, which could further stimulate microhomology annealing. Alternatively, short 3′ ssDNA ends in *sgs1*Δ *exo1*Δ may be more stable, or short ssDNA could limit loading of Rad51 that is known to inhibit recombination between short homologous sequences or microhomologies (e.g. (41,54)).

In summary, the microhomology at the junctions and the genetic requirements for the foldback inversions described here support a model in which the two ends of a single DSB engage in distinct repair pathways. One end primes DNA synthesis at microhomology, while the other end captures a duplicated region by NHEJ or Alt-EJ (Figure 1C). The model suggesting ligation of ssDNA and dsDNA ends by NHEJ is supported by NHEJ-dependent capture of ssDNA oligonucleotides at nuclease induced DSBs in yeast and humans (3,22); however, we cannot exclude the possibility that second strand is synthesized prior to ligation. It will be of great interest to test the joining of ssDNA and dsDNA ends in *in vitro* reconstituted NHEJ in the presence of Pol4 in future studies. Our analysis focused on events with >5-nt inversions, but it is highly likely that inversions as short as 1–4 bp are formed by a similar mechanism. These shorter inversions are difficult to distinguish from typical NHEJ errors associated with deletions. To investigate them further, we compared the read number of the sequence corresponding to foldback-mediated synthesis of only 2 nt (‘TT’) in wild-type and mutant strains with higher or lower numbers of foldback inversions. We found that the read number was higher in *sgs1*Δ *exo1*Δ, *rfa1*-t33, or *rtt105*Δ mutants and absent in *pol32*Δ, *mre11*Δ or *lig4*Δ mutants. This suggests that the mechanism described here can lead to foldback inversions of just 2 nt (Figure 2D).

### Rad27, RPA and extensive resection suppress distant templated insertions

**Figure 3.**
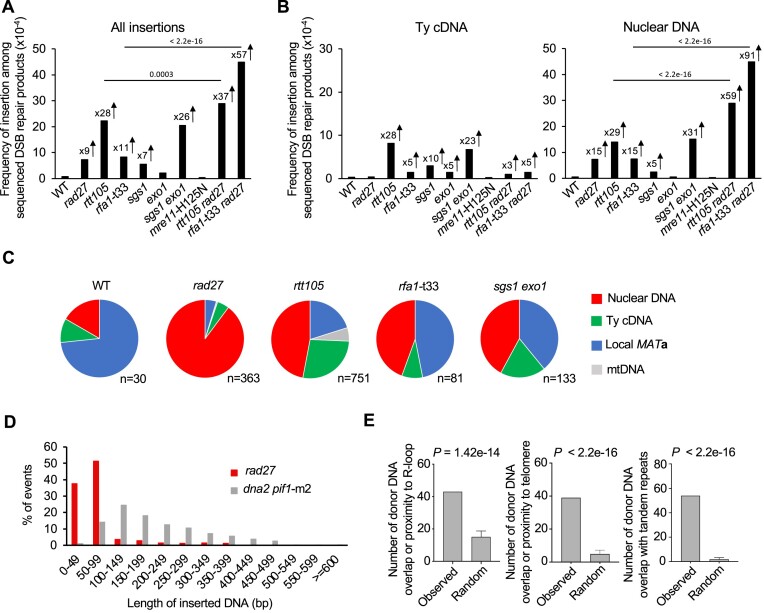
RPA and Rad27 suppress distant templated insertions **(A, B)** Analysis of templated insertions in indicated mutants. Change in frequency of all insertions **(A)** and insertion of Ty cDNA or nuclear genome **(B)** among sequenced DSB repair products are shown. The insertions from *MAT***a** locus are excluded. *P* values were determined using χ^2^ test; n – number of DSB repair products tested for each mutant is shown in Supplementary Table S2. **(C)** Pie charts representing distribution of insertion donor DNA in indicated mutants. **(D)** Size distribution of DNA inserted in *rad27Δ* mutant and *dna2Δ pif1*-m2 mutant. The data of *dna2Δ pif1*-m2 mutant are taken from a previous publication (9). Complex insertions and *MAT***a** insertions were not included in the analysis. **(E)** Feature analysis of inserted DNA in *rad27Δ* mutant. *P*-values were calculated using a one-sided permutation test. Proximity is defined as a sequence within 0.2 kb from the R-loop, within 1 kb from telomere and the overlap with tandem repeats indicates at least 1 bp overlap.

In addition to short local inverted templated insertions, we also observed distant templated insertions from all over the genome, as previously shown in Dna2 deficient cells (3,9). Besides the mutants tested above, we also looked in *rad27*Δ as it processes 5′ flaps during lagging strand synthesis like Dna2 (e.g. (24,55)). Templated insertions from across the genome increased by approximately 10–30-fold in *rfa1*-t33, *rtt105*Δ, *rad27*Δ, and *sgs1*Δ *exo1*Δ mutants, but not in *sgs1*Δ, *exo1*Δ, or *mre11*-H125N mutants (Figure 3A–C, Supplementary Table S2). Interestingly, Sgs1 and Rtt105 were found to suppress insertions from Ty cDNA, which might be related to their role in controlling Ty transposition (56,57). We have plotted insertions of Ty1 cDNA from *rtt105*Δ that showed the highest number of these events (*n* = 98). As previously observed, most insertions originate from 3′ LTR region (supplementary Figure S2A) (8,58).

The *rad27*Δ cells exhibited a 15-fold increase in insertions from the nuclear genome. This demonstrates the much higher sensitivity of *Break-Ins* analysis compared to previous analyses of a limited number of individual NHEJ products, which showed no insertions greater than 10 bp in *rad27Δ* cells (3). Unlike in Dna2 deficient cells, very few insertions originated from rDNA (Figure 3B, Supplementary Table S2). These insertions are templated (copy-paste), as we observed the expected number of insertions originating from essential genes (Supplementary Figure S2B). Deletions within essential genes are expected to be lethal and, therefore, not detected by *Break-Ins*. Nearly all inserted DNA fragments are shorter than 100 bp, with a median size of 88 bp, and about 40% are shorter than 50 bp. This contrasts with *dna2*Δ mutants, in which most insertions (>85%) are longer than 100 bp, with a median size of 187 bp (Figure 3D). The size of inserted DNA in *rad27*Δ corresponds to the size of 5′ flaps observed in *rad27*Δ cells by electron microscopy (24). Thus, alternative processing of 5′ flaps left in *rad27*Δ cells may be the source of fragments that are inserted at DSBs. Analysis of 310 insertions in *rad27*Δ cells originating from throughout the genome shows that inserted DNA often overlaps with microsatellite DNA (1–12 nt repeat) (Figure 3E) or comes from within or next to telomeric repeats. Rad27 activity is important for maintaining the stability of telomeric repeats and microsatellite DNA sequences (59–61), indicating that the inserted DNA might originate from loci most vulnerable to replication problems in *rad27*Δ. Three examples of the hotspots of the inserted DNA are shown (Supplementary Figure S2C). Inserted DNA also originated from previously mapped R-loops, consistent with the possible role of Rad27 in genome stability at R-loop generating loci (62).

We identified a combined total of 321 insertions from the nuclear genome in *rtt105*Δ and *rfa1*-t33 mutants, and these insertions also originated preferentially from fragile and repetitive sites of the genome (see Supplementary Figure S3). We note that 45 insertions from *sgs1*Δ *exo1*Δ were not sufficient to establish the features of DNA inserted from the genome. Furthermore, the double mutants *rfa1*-t33 *rad27*Δ or *rtt105*Δ *rad27*Δ showed an additive effect on insertion level, suggesting that RPA and Rad27 operate in different pathways to prevent templated insertions at DSBs. In conclusion, templated insertions are common in mutants defective in nucleases processing 5′ flaps, Rad27 or Dna2, as well as in RPA mutants.

### Distant inverted repeats are increased in *rad27*Δ and RPA mutants

**Figure 4.**
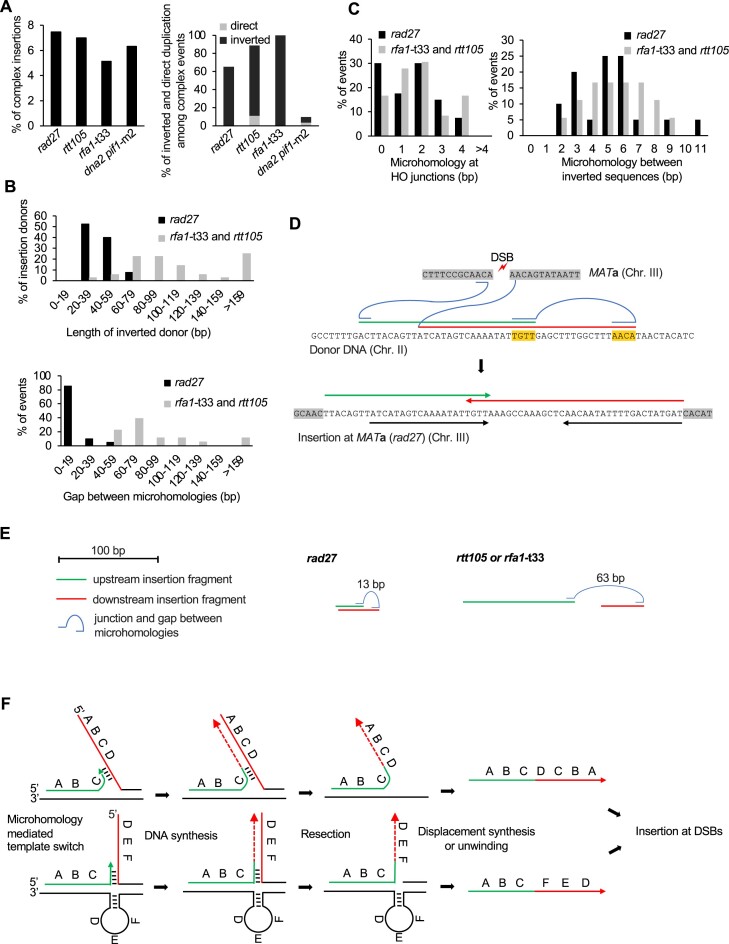
Distant inverted insertions in *rad27Δ* and RPA mutants **(A)** Percentage of complex insertions among all insertions (left panel) and percentage of two sequence insertions from the same locus in inverted or direct orientation among complex insertions in indicated mutants (right panel, rDNA and Ty sequences are excluded). **(B)** Size analysis of insertion donor DNA (upper panel) and the size of the gap between two inverted microhomologous sequences (lower panel). **(C)** Microhomology analysis between inserted DNA and DSB ends (left) and microhomology between two inserted fragments (right). **(D)** An example of inverted insertion from *rad27*Δ cells. The upper panel shows the *MAT***a** sequence and two donor sequences inserted at DSB. Curves mark the junctions, and the short horizontal lines represent the microhomology used for junctions. Microhomology between inverted sequences is highlighted. Final repair product carrying two inserted DNA fragments is shown below. The two black arrows represent inserted identical sequences with opposite orientations. **(E)** Scheme of the examples of inverted insertion events from *rad27*Δ and RPA mutants. The size of the gap between two inverted microhomologous sequences is indicated. **(F)** A model explaining distant inverted insertion at DSB. During replication in *rad27*Δ or RPA mutants, 5′ flaps or secondary structures can accumulate, respectively. Template switch at microhomologies initiates formation of inverted sequences. Upon resection and strand displacement, the inverted fragment is captured at DSB via NHEJ.

Analysis of templated insertion events in *rad27*Δ, *rtt105*Δ, *rfa1*-t33 and *dna2*Δ *pif1*-m2 mutants shows that 5–7% of them are complex, carrying at least two fragments (Figure 4A). A frequent pattern of complex insertions was observed in *rad27*Δ (65.2%, 15/23), in which two fragments originate from the same locus but in inverted orientation (Figure 4A). Very rare complex insertions involving two sequences from a single locus inserted in the same orientation were observed. The inverted sequences had partially overlapped sequences with a median length of 50 bp and a distance of ∼2–20 bp between microhomology junctions. We further found that similar double inverted insertions were also observed in *rtt105*Δ (78% of complex events, 14/18), *rfa1*-t33 (100%, 3/3) and less frequently in Dna2 deficient cells (6%, 9/144). The reason for lower levels of inverted insertions of two sequences from the same locus in *dna2*Δ is not known. In *rtt105*Δ mutants, the size of inverted sequences was longer (median = 150 bp), and the two inverted sequences from a single locus are rather adjacent to each other with little or no overlap (distance between junctions was between 30 and 119 bp). Microhomologies between the entire complex insertion and DSB ends are typical for NHEJ (median = 2 bp, range of 0–4 bp), while microhomology between the two inverted sequences is longer (median = 6 bp, range of 2–11), suggesting a mechanism other than NHEJ generating the inversion itself (Figure 4B,C). An example of sequences and the structure of the double inverted insertion are shown (Figure 4D–E, Supplementary Figure S4). Taken together, we propose a model that involves both a microhomology-mediated DNA synthesis step to generate inversion and a NHEJ step that generates insertion (Figure 4F). First, microhomology-mediated DNA synthesis occurs at short 5′ flaps (*rad27*Δ) or in the context of secondary structures formed in cells with limited RPA, leading to inversions. The longer microhomology at the junction between inverted fragments is consistent with possible involvement of microhomology-mediated DNA synthesis. Displacement of a newly synthesized strand with inverted sequences is followed by its insertion at the DSB and results in distant inverted insertion.

### Inversions can form within extrachromosomal pieces of ssDNA

**Figure 5.**
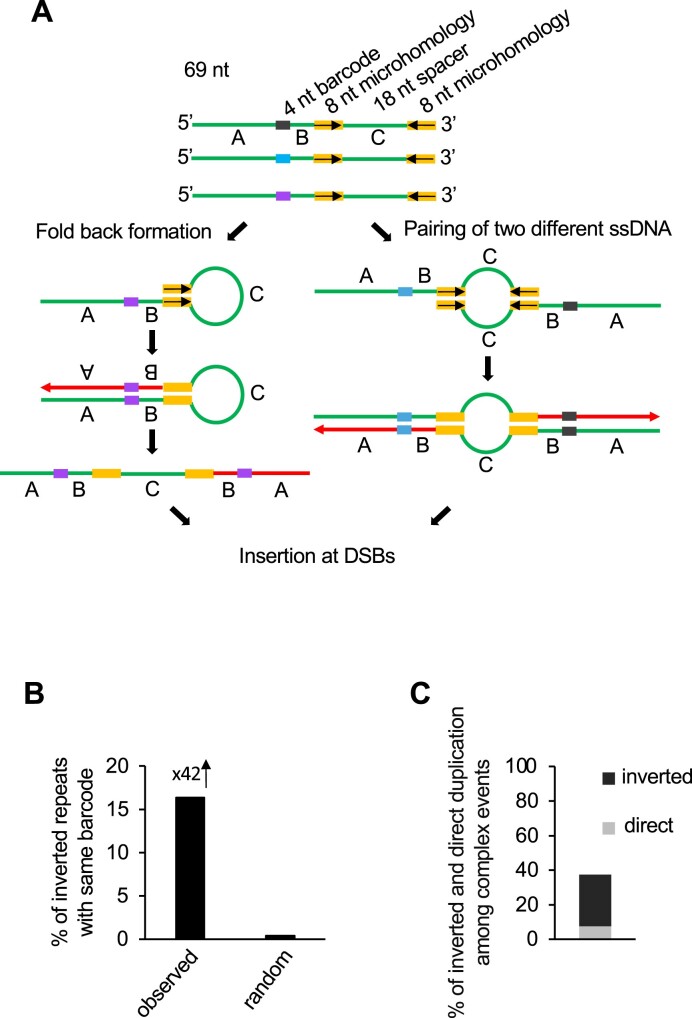
ssDNA can generate foldback inversions at DSBs **(A)** The experimental system to test insertions of transformed ssDNA. The 69-nt ssDNA contains a 4-nt barcode in the middle, two 8-nt inverted microhomologies and an 18-nt spacer. The presence of barcodes within ssDNA allows differentiation between fold-back inversion (two identical barcodes) and pairing between two different ssDNA molecules (two different barcodes). **(B)** Bar graph shows random and observed percentage of inversions with identical barcode. **(C)** Number of complex insertions carrying two fragments from the same locus in inverted or direct orientation from wildtype stationary phase cells. The sequencing reads of wildtype stationary phase cells are taken from a previous publication (9).

To test directly whether such a mechanism is possible, we transformed ssDNA of 69 nt carrying an 8-nt long internal inverted microhomology into wild-type cells followed by plating cells on YEP-Gal media to induce DSB at *MAT***a** locus (Figure 5A). Products of DSB repair with or without insertion of transformed fragments were collected and sequenced as described above. To distinguish between inversions generated by two molecules and those within a single molecule, we added random internal 4-nt barcodes to the transformed DNA. Inversions formed within a single molecule would carry two identical barcodes, while inversions formed between two molecules would carry two different barcodes (Figure 5A), with 4-nt barcodes providing 256 possible combinations.

*Break-Ins* analysis after the transformation of such ssDNA demonstrated a significant number of inverted insertions with barcodes (*n* = 342), 16% of which were marked by identical barcodes. This is a much higher number than expected by chance (<1%) (Figure 5B). This result supports the possibility that inversions can occur within ssDNA, followed by its insertion at the DSBs by NHEJ.

### Distant inverted repeats in stationary cells

We documented templated inverted insertions in mutants deficient in DNA replication and repair. To check whether similar events can occur in wild-type cells, we examined sequences of previously published templated insertions from stationary phase cells (9). In wild-type stationary phase cells, templated insertions are far more common than in growing cells. While most of the insertions originate from mtDNA and Ty cDNA, stationary phase cells at 16 days exhibited ∼300-fold increase of templated insertions from all over the genome. About 12% of these insertions are complex events carrying two fragments, and among these events with two donors from nuclear genome (rDNA, Ty excluded), we found 30% carried sequences from the same locus and in inverted orientation. Therefore, distant inverted insertions can occur even in wild-type cells in suboptimal conditions (Figure 5C).

## Discussion

Templated insertions can occur at programmed breaks at the V(D)J locus or at endonuclease induced breaks (3,6,21,63). Despite their prevalence, the mechanisms underlying such events remain poorly understood, and the genetic pathways regulating these events are largely unknown. In this study, we utilized a yeast model organism and the *Break-Ins* method to sequence hundreds of thousands of DSB repair products. Through this approach, we identified mutants with increased levels of simple and complex, local and distant templated insertions. Additionally, we have elucidated two mechanisms leading to the formation of either local or distant inversions.

In both wild type and most mutants tested, we observed local templated insertions that likely proceed through a foldback inversion mechanism mediated by a short inverted microhomology located immediately next to the DSB. These foldback inversions ranged from 2 to approximately 150 bp in length, with the majority being under 14 bp. The requirements for Polδ and NHEJ, as well as analysis of microhomologies at insertion junctions suggests a hybrid mechanism in which one end primes DNA synthesis at local microhomology while the other end uses NHEJ to capture newly synthesized DNA. As a result, longer inverted repeats are formed (Figure 1C). The DNA synthesis step of the proposed mechanism shares some features typical for MMBIR: (i) synthesis is initiated at microhomology, (ii) newly synthesized DNA is unwound from the template and (iii) a polymerase essential for break-induced replication (BIR) is likely responsible for the DNA synthesis step. In MMBIR, unwinding could lead to additional cycles of template switch and DNA synthesis (4), whereas here, the unwound strand is simply joined with the second end of the break by NHEJ or, rarely, by Alt-EJ (4). In humans, POLQ-mediated Alt-EJ is significantly more efficient than in yeast lacking POLQ, suggesting a potentially more prominent role in templated insertions. However, POLQ by itself predominantly generates shorter templated insertions, typically up to ∼30 nt in length (17,18). Moreover, studies have shown that POLQ is dispensable for templated insertions at CRISPR/Cas9 nuclease-induced DSBs (6). A comparable hybrid DSB repair to the one described here was previously observed in experimental systems and humans, but it is initiated by homology driven strand invasion and DNA synthesis followed by second end capture by end joining with microhomology of 1–5 bp (e.g. (64)). Local templated insertions were found to be limited by RPA, likely due to its known inhibitory role in the annealing of microhomologies (29–33). Lower levels of RPA also affected the occurrence of distant templated insertions from various fragile and repetitive regions of the genome, which could be related to RPA’s role in preventing secondary structure formation and regulation of a broad range of DNA nucleases (e.g. (65–67)). Cancer cells often experience a shortage of RPA due to replication stress and excessive firing of replication origins (34,35). Therefore, microhomology-mediated templated insertions in cancer cells could be partially attributed to insufficient RPA to protect all ssDNA. Extensive resection also suppresses foldback inversion, which could be related to reduced RPA or Rad51 binding to very short ssDNA, both of which are known to limit microhomology-mediated repair. Previous studies have shown that extensive resection is important to prevent *de novo* telomere formation at DSBs, possibly by limiting binding of the Cdc13–Stn1–Ten1 complex (68,69), a complex structurally similar to RPA.

We have also observed an increased level of templated insertions from various genomic locations in *rad27*Δ cells. These insertions were shorter than those observed in any other mutant tested here and often contained microsatellite, telomeric DNA, and other repetitive regions. Rad27, the 5′ flap endonuclease (human FEN1), plays a crucial role in replication and stability at these loci (59–61,70,71). Thus, the pattern of templated insertions and the origin of inserted DNA closely align with the functions of Rad27. Notably, the most significant increase in templated insertions among mutants tested so far is observed in another 5′ flap endonuclease mutant, *dna2*Δ (3,9,22). DNA2 nuclease is commonly overexpressed in various cancer types, and this elevated level of DNA2 is often essential for the survival of cancer cells (72–75). This suggests that the number of substrates processed by DNA2 increases in cancer cells, and the high number of templated insertions observed could be related to these substrates.

In RPA mutants (*rtt105*Δ or *rfa1*-t33) and in *rad27*Δ mutants, we observed a common pattern of complex templated insertions consisting of two sequences from the same locus but in inverted orientation. Notably, in *rad27*Δ mutants the inverted sequences overlap with each other, thus forming inverted repeats, whereas in RPA mutants they mostly do not overlap. Analysis of previously published complex templated insertions from Dna2 deficient cells or even from wild-type stationary phase cells also showed cases of both overlapping and non-overlapping inverted sequences. We propose that such inversions arise through microhomology-mediated template switch within ssDNA intermediates of replication, followed by displacement of the inverted sequence and its insertion at the DSB (model depicted in Figure 4F). Alternatively, inversions can occur within extrachromosomal DNA. Supporting this possibility, extrachromosomal transformed ssDNA carrying internal inverted microhomologies forms such inversions at DSBs with high frequency. The presence of longer microhomology between two inverted sequences supports the possibility that DNA synthesis was involved in its formation. Recently, the formation of extrachromosomal DNA with inverted DNA that reintegrates into the genome was postulated to explain the formation of inversions (19). Like the model proposed here, inverted sequences form through template switching at microhomologies during replication followed by strand displacement. It will be of great interest to investigate whether distant inverted templated insertions, as described here, can also occur in human cancer genomes. The high level of inverted microhomologies suggests that such events can occur nearly anywhere in human or other genomes (76,77). Once formed, overlapping inverted sequences (inverted repeats) can give rise to secondary structures within the genome, thereby promoting additional genome instabilities (39,76,78–88). Altogether, this work provides insights into the origin of templated insertions that can be tested in cancer cells in future.

## Supplementary Material

gkae1159_Supplemental_File

## Data Availability

The *Break-Ins* data has been archived at GEO under the accession number GSE260753. All original code has been deposited on GitHub. For local inversion insertion from *MAT***a**, the corresponding code is available at: https://github.com/gucascau/iMMBIR.git. The codes utilized for characterizing inverted repeats resulting from ssDNA transformation have been made available at: https://github.com/gucascau/iDSBInvert.git. The code for large insertion detection can be accessed at the following GitHub repository: https://github.com/gucascau/iDSBins.git. The features related to large insertion analysis have been uploaded to a dedicated GitHub repository: https://github.com/gucascau/LargeInsertionFeature.git. Data are also available at https://figshare.com/s/77a49adc36a3f6159096. Any additional information required to reanalyze the data reported in this paper is available from the lead contact upon request.
