# Supplementary material for: RPA and Rad27 limit templated and inverted insertions at DNA breaks
Source: Nucleic Acids Res. 2024 Dec 3;53(1):gkae1159. doi: 10.1093/nar/gkae1159 (PMC11724301; doi:10.1093/nar/gkae1159)
Supplement: gkae1159_Supplemental_File [file gkae1159_supplemental_file.pdf]

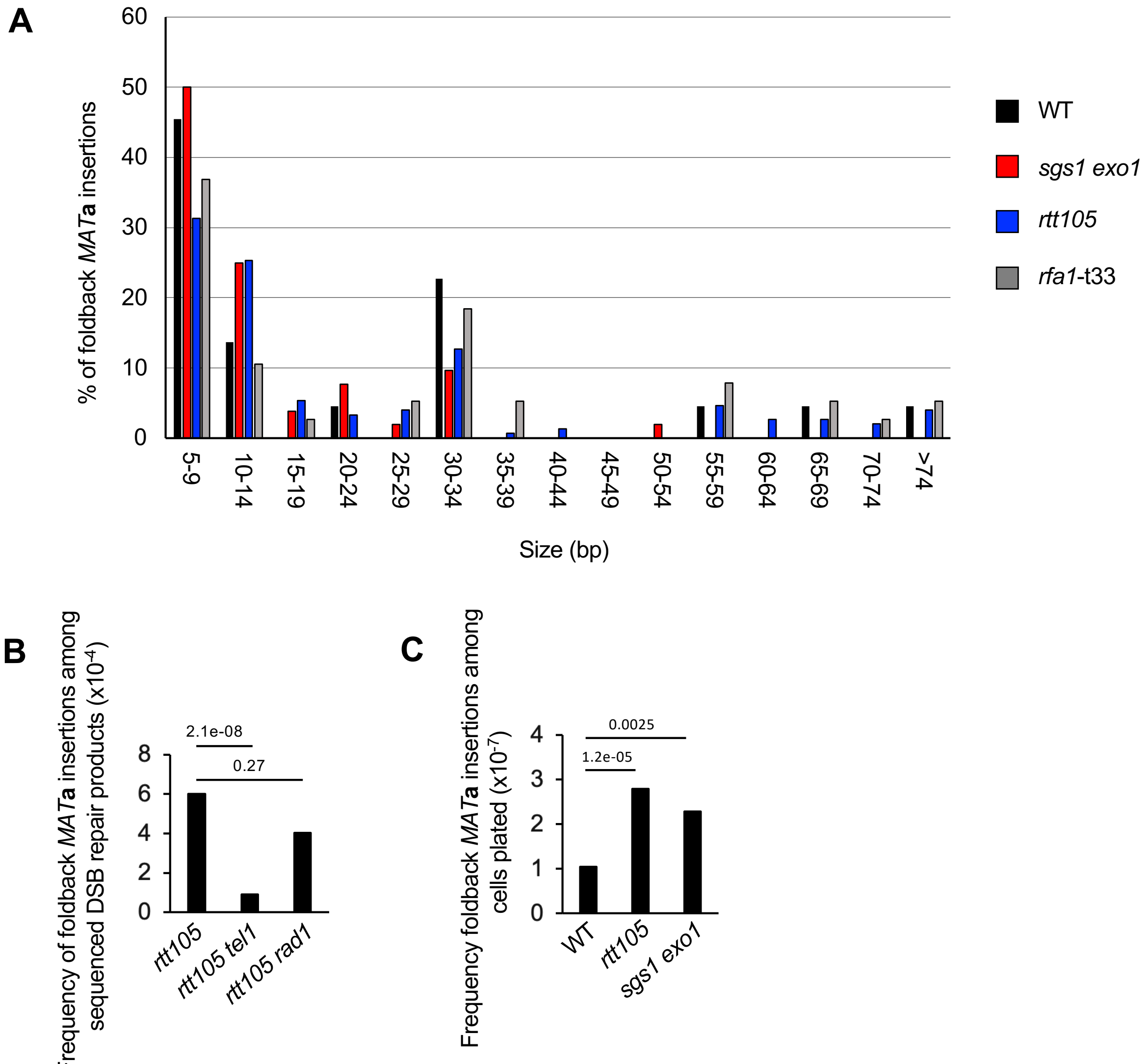
A
% of foldback MATa insertions
60
50
40
30
20
10
0
5-9
10-14
15-19
20-24
25-29
30-34
35-39
40-44
45-49
50-54
55-59
60-64
65-69
70-74
>74
Size (bp)
WT
sgs1 exo1
rtt105
rfa1-t33
B
Frequency of foldback MATa insertions among sequenced DSB repair products (x10-4)
2.1e-08
0.27
8
6
4
2
0
rtt105
rtt105 tel1
rtt105 rad1
C
Frequency foldback MATa insertions among cells plated (x10-7)
0.0025
1.2e-05
4
3
2
1
0
WT
rtt105
sgs1 exo1

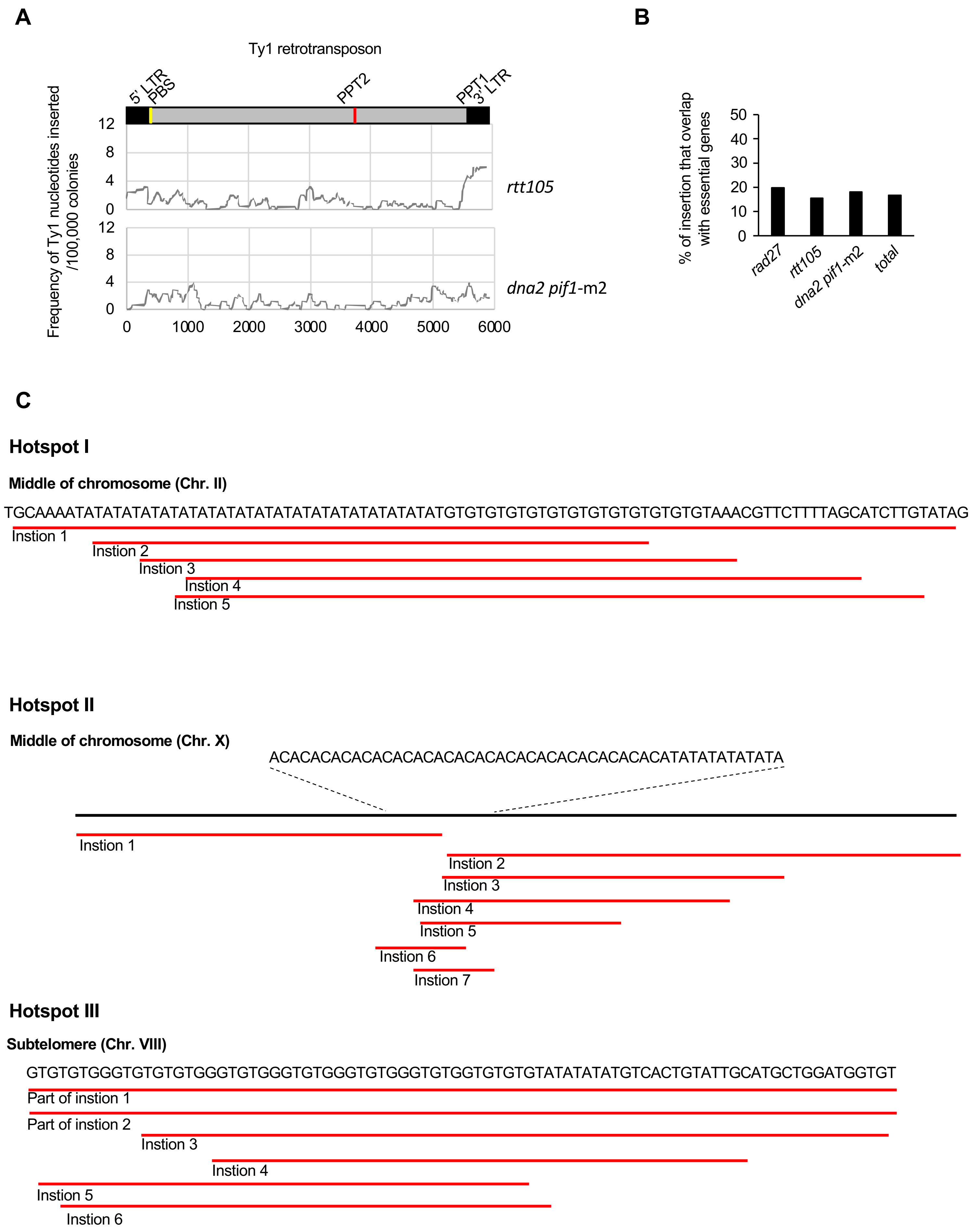
A
Ty1 retrotransposon
5' LTR
PBS
PPT2
PPT1
3' LTR
Frequency of Ty1 nucleotides inserted /100,000 colonies
12
8
4
0
rtt105
dna2 pif1-m2
0
1000
2000
3000
4000
5000
6000
B
% of insertion that overlap with essential genes
50
40
30
20
10
0
rad27
rtt105
dna2 pif1-m2
total
C
Hotspot I
Middle of chromosome (Chr. II)
TGCAAAATATATATATATATATATATATATATATATATATATATATATATGTGTGTGTGTGTGTGTGTGTGTGTAAACGTTCTTTTAGCATCTTGTATAG
Instion 1
Instion 2
Instion 3
Instion 4
Instion 5
Hotspot II
Middle of chromosome (Chr. X)
ACACACACACACACACACACACACACACACACACACACACATATATATATATA
Instion 1
Instion 2
Instion 3
Instion 4
Instion 5
Instion 6
Instion 7
Hotspot III
Subtelomere (Chr. VIII)
GTGTGTGGGTGTGTGTGGGTGTGGGTGTGGGTGTGGGTGTGGTGTGTGTATATATATGTCACTGTATTGCATGCTGGATGGTGT
Part of instion 1
Part of instion 2
Instion 3
Instion 4
Instion 5
Instion 6

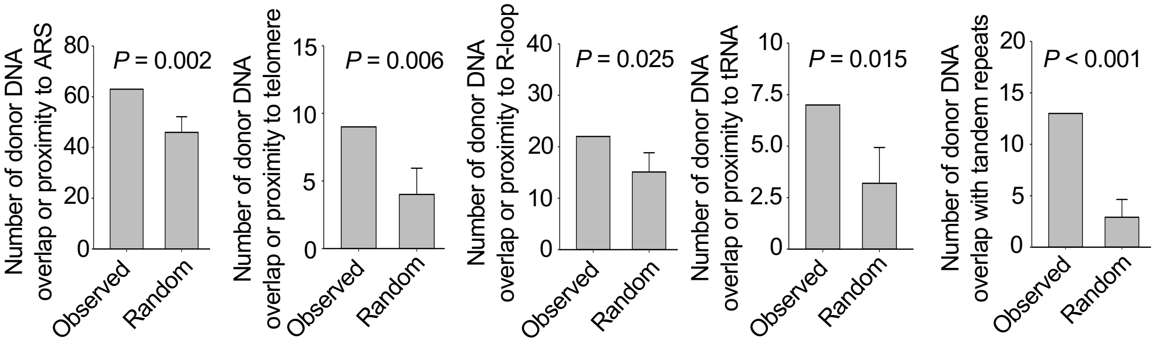
Number of donor DNA overlap or proximity to ARS
P = 0.002
80
60
40
20
0
Observed
Random
Number of donor DNA overlap or proximity to telomere
P = 0.006
15
10
5
0
Number of donor DNA overlap or proximity to R-loop
P = 0.025
40
30
20
10
0
Number of donor DNA overlap or proximity to tRNA
P = 0.015
10
7.5
5.0
2.5
0
Number of donor DNA overlap with tandem repeats
P < 0.001
20
15
10
5
0

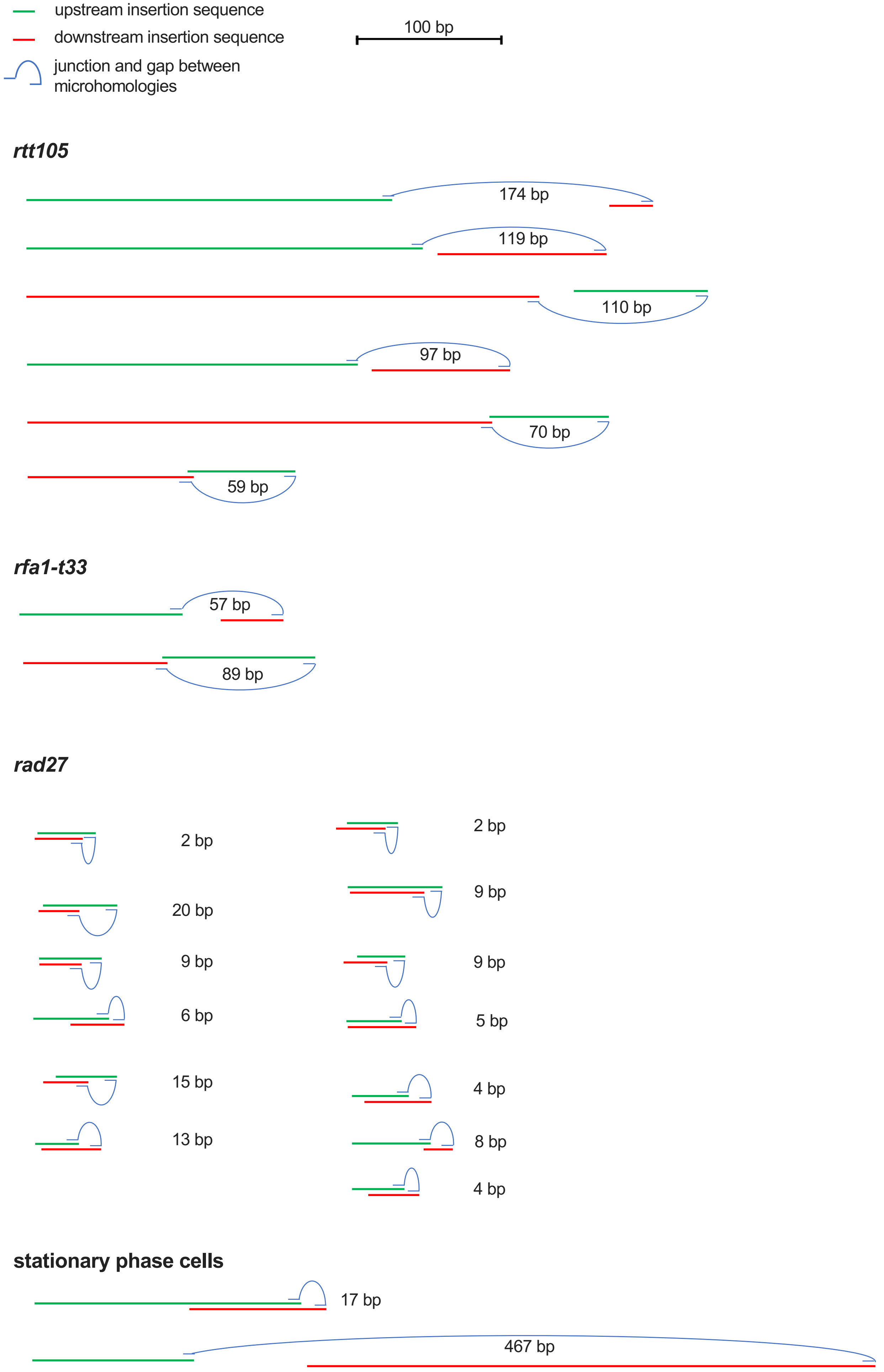
upstream insertion sequence
downstream insertion sequence
junction and gap between microhomologies
100 bp
rtt105
174 bp
119 bp
110 bp
97 bp
70 bp
59 bp
rfa1-t33
57 bp
89 bp
rad27
2 bp
2 bp
9 bp
20 bp
9 bp
9 bp
6 bp
5 bp
15 bp
4 bp
13 bp
8 bp
4 bp
stationary phase cells
17 bp
467 bp

Supplementary Figure Legends

Supplementary Figure 1. Analysis of local inverted insertions

(A) Size analysis of the foldback inversions from wild-type and indicated mutants. Foldback inversions shorter than 5 bp were not included.

(**B**) Analysis of foldback inversions in *rtt105*Δ and indicated derivative mutants. Foldback inversions shorter than 5 bp were not included. P values were determined using χ^2 test; n – number of DSB repair products tested for each mutant is shown in Table S2.

(C) Analysis of foldback inversions in wild-type and indicated mutants among cells plated and normalized by viability. Foldback inversions shorter than 5 bp were not included. P values were determined using χ^2 test; n – number of DSB repair products tested for each mutant is shown in Table S2.

Supplementary Figure 2. Analysis of inserted DNA

(A) Analysis of Ty1 sequences inserted at DSBs. Frequency of each Ty1 nucleotide insertion per 10^6 of DSB repair products in *rtt105*Δ and *dna2*Δ *pif1*-m2 mutant cells. Schematic of Ty1 shown at the top indicating long terminal repeats (LTR), polypurine tracts (PPT1/2), and primer binding site (PBS).

(**B**) The percentage of insertion overlapping with essential genes in indicated mutants and the percentage of essential genes in yeast genome (total).

(C) The hotspots of inserted DNA from repetitive regions of the genome. The red lines indicate different inserted sequences from the same locus.

Supplementary Figure 3. Feature analysis of inserted DNA in *rtt105*Δ mutant and *rfa1*-t33 mutants. The data of *rtt105*Δ mutant and *rfa1*-t33 mutant was combined for this analysis. P-values were calculated using a one-sided permutation test. Proximity is defined as a sequence within 0.2 kb from the R-loop or tRNA, within 1 kb from ARS or telomere, and the overlap with tandem repeats indicates at least 1 bp overlap.

Supplementary Figure 4. Scheme of the examples of inverted insertion events from *rad27*Δ, *rtt105*Δ, *rfa1*-t33 mutant and wildtype stationary phase cells. The size of the gap between two inverted microhomologous sequences is marked.

Table S1. List of strains used in this study.

Strain name	Parental strain	Genotype	Source
JKM139		DELho *hml*::*ADE1 MAT***a** *hmr*::*ADE1 ade1 leu2-3,112 lys5 trp1*::*hisG ura3-52 ade3*::*GAL10*::*HO*	(1)
yYY398	JKM139	*rad27::klTRP1*	(2)
yYY591	JKM139	*rtt105::hphMX*	this study
yYY616	JKM139	*rfa1-t33*	this study
yYY596	JKM139	*rtt105::hphMX rad27::klTRP1*	this study
yYY711	JKM139	*rtt105::hphMX tel1::TRP1*	this study
yYY712	JKM139	*rtt105::hphMX rad1::klTRP1*	this study
yYY643	JKM139	*rfa1-t33 rad27::klTRP1*	this study
yWH475	JKM139	*dna2::kanMX pif1*-m2	(3)
yGI199	JKM139	*sgs1::KanMX exo1::TRP1*	(3)
yGI200	JKM139	*sgs1::KanMX*	(3)
yGI198	JKM139	*exo1::TRP1*	(3)
yZZ540	JKM139	*mre11-H125N::URA3*	(4)
yYY592	JKM139	*mre11::hphMX*	this study
yYY361	JKM139	*rev3::klTRP1*	this study
yYY590	JKM139	*rad30::kanMX*	this study
yYY387	JKM139	*pol32::natMX*	(5)
yYY119	JKM139	*yku70::TRP1*	this study
yYY399	JKM139	*lig4::klTRP1*	(5)
yYY400	JKM139	*pol4::klTRP1*	(5)

1. Moore, J.K. and Haber, J.E. (1996) Cell cycle and genetic requirements of two pathways of nonhomologous end-joining repair of double-strand breaks in Saccharomyces cerevisiae. *Mol Cell Biol*, **16**, 2164-2173.
2. Yu, Y., Pham, N., Xia, B., Papusha, A., Wang, G., Yan, Z., Peng, G., Chen, K. and Ira, G. (2018) Dna2 nuclease deficiency results in large and complex DNA insertions at chromosomal breaks. *Nature*, **564**, 287-290.
3. Zhu, Z., Chung, W.H., Shim, E.Y., Lee, S.E. and Ira, G. (2008) Sgs1 helicase and two nucleases dna2 and exo1 resect DNA double-strand break ends. *Cell*, **134**, 981-994.
4. Shim, E.Y., Chung, W.H., Nicolette, M.L., Zhang, Y., Davis, M., Zhu, Z., Paull, T.T., Ira, G. and Lee, S.E. (2010) Saccharomyces cerevisiae Mre11/Rad50/Xrs2 and Ku proteins regulate association of Exo1 and Dna2 with DNA breaks. *EMBO J*, **29**, 3370-3380.
5. Yu, Y., Wang, X., Fox, J., Yu, R., Thakre, P., McCauley, B., Nikoloutsos, N., Li, Q., Hastings, P.J., Dang, W. *et al.* (2023) Yeast EndoG prevents genome instability by degrading cytoplasmic DNA. *bioRxiv*.

Supplementary Table S2. Frequency and types of DNA insertions

							donor						frequency per 10^4 DSB repair products						
**strain name**	**genotype**	**number of colonies**	**number of colonies normalized by HO cut (n)**	**number of cells plated**	**all insertions excluding *MAT*a**	**complex insertions**	***MAT*a***	**mtDNA**	**Ty DNA**	**all nuclear DNA**	**rDNA**	**nuclear w/o rDNA**	**all insertions excluding *MAT*a**	***MAT*a***	**mtDNA**	**Ty DNA**	**all nuclear DNA**	**rDNA**	**nuclear w/o rDNA**
JKM139	WT**	102976	101711	210000000	8	0	22	0	3	5	2	3	0.8	2.2	0.0	0.3	0.5	0.2	0.3
yYY398	*rad27*	600672	445695	730000000	323	25	17	2	18	326	16	310	7.2	0.4	0.0	0.4	7.3	0.4	7.0
yYY591	*rtt105*	374304	251798	720000000	560	40	151	42	205	353	63	290	22.2	6.0	1.7	8.1	14.0	2.5	11.5
yYY616	*rfa1* -t33	53184	48257	360000000	40	3	38	0	7	36	5	31	8.3	7.9	0.0	1.5	7.5	1.0	6.4
yYY596	*rtt105 rad27*	111840	103358	360000000	299	11	27	1	10	299	16	283	28.9	2.6	0.1	1.0	28.9	1.5	27.4
yYY643	*rfa1* -t33 *rad27*	14976	14254	240000000	64	3	6	1	2	64	2	62	44.9	4.2	0.7	1.4	44.9	1.4	43.5
yWH475	*dna2 pif1* -m2**	197264	178507	1120000000	3966	250	31	0	255	3963	625	3343	222.2	1.7	0.0	14.3	200.9	31.7	187.0
yGI199	*sgs1 exo1*	55000	37156	440000000	76	4	52	0	25	56	11	45	20.5	14.0	0.0	6.7	15.1	3.0	12.1
yGI200	*sgs1*	106080	75160	200000000	42	1	12	3	22	18	3	15	5.6	1.6	0.4	2.9	2.4	0.4	2.0
yGI198	*exo1*	54240	41429	120000000	9	0	11	1	6	2	0	2	2.2	2.7	0.2	1.4	0.5	0.0	0.5
yZZ540	*mre11*- H125N	144320	142941	200000000	6	1	11	0	3	4	1	3	0.4	0.8	0.0	0.2	0.3	0.1	0.2
yYY361	*rev3*	64160	60274	200000000	4	0	8	0	3	1	0	1	0.7	1.3	0.0	0.5	0.2	0.0	0.2
yYY590	*rad30*	37296	32924	120000000	0	0	9	0	0	0	0	0	0.0	2.7	0.0	0.0	0.0	0.0	0.0
yYY387	*pol32*	44160	39989	120000000	2	0	0	0	1	1	0	1	0.5	0.0	0.0	0.3	0.3	0.0	0.3
yYY711	*rtt105 tel1*	79360	78245	110000000	18	0	7	1	7	11	1	10	2.3	0.9	0.1	0.9	1.4	0.1	1.3
yYY712	*rtt105 rad1*	29280	24893	120000000	3	1	10	0	0	4	0	4	1.2	4.0	0.0	0.0	1.6	0.0	1.6
yYY592	*mre11*	52176	10892	1800000000	0	0	0	0	0	0	0	0	0	0***	0	0	0	0	0
yYY119	*yku70*	46960	1025	2400000000	0	0	2	0	0	0	0	0	0	8.3E-10***	0	0	0	0	0
yYY399	*lig4*	65280	1223	2400000000	0	0	1	0	0	0	0	0	0	4.2E-10***	0	0	0	0	0
yYY400	*pol4***	173760	140327	2400000000	1	0	48	0	0	1	0	1	0.1	2.0E-8***	0	0	0.1	0	0.1

* indicates the local foldback inversions from *MAT* **a** locus related to Figure 1 and Figure 2

** indicates data from Yu Y *et al* (9)

*** indicates the frequency per cells plated
